# Spatial distribution of soil nutrients and evaluation of cultivated land in Xuwen county

**DOI:** 10.7717/peerj.13239

**Published:** 2022-06-30

**Authors:** Chao Zheng, Xiaofei Yang, Zhiqiang Liu, Kexing Liu, Yongxiang Huang

**Affiliations:** 1 College of Chemistry and Environment, Guangdong Ocean University, Zhanjiang, China; 2 Centre Testing International Pinbiao (Guangzhou) Co., Ltd., Guangzhou, China; 3 College of Natural Resources and Environment, South China Agricultural University, Guangzhou, China; 4 College of Coastal Agricultural Sciences, Guangdong Ocean University, Zhanjiang, China

**Keywords:** Soil nutrients, Geostatistics, Spatial variability, Soil fertility

## Abstract

It is of great significance to promote the quantitative research of soil science and the implementation of precision agriculture. On this basis, taking Xuwen County as the research object, this paper comprehensively analyzed the soil characteristics of cultivated land in Xuwen County and clarify the soil nutrient content and spatial distribution characteristics of cultivated land in Xuwen County, this paper comprehensively applied the methods of geostatistics, geographic information system (GIS) and fuzzy mathematics, and referred to the cultivated land quality grade standard (GB/T 33469-2016), to analyze the soil characteristics and evaluate the soil fertility of this region. The results show that the optimal interpolation model of soil pH and available phosphorus (AP) is a Gaussian model, and the optimal interpolation model of soil organic matter (SOM), available nitrogen (AN) and available potassium (AK) is a J-Bessel model. In addition, the spatial correlation of AK is weak, whereas pH, SOM, AN and AP show moderate spatial correlation. The proportion of excellent, good, average, medium and poor comprehensive fertility index are 26.00%, 32.67%, 19.33%, 19.00% and 3.00%, respectively. The overall level of soil fertility in Xuwen County is above the average, and the fertility quality presents an obvious trend of high in the South and low in the North. Areas that above average fertility are mainly distributed in Maichen Town, Qujie Town, Nanshan Town and Chengbei Town. The results can provide theoretical basis for improving the utilization rate of chemical fertilizer, fine management of cultivated land and ecological environment in this region, which can help in decision-making of precision fertilization.

## Introduction

The soil organic matter and nutrients are the basis of land productivity, necessary condition of plant growth, and one of the important indicators to measure soil fertility (Mamuye et al., 2021), but little is known about the spatial distribution of soil organic matter and nutrients. Many factors can influence the spatial distribution of SOM and soil nutrients in an orchard, including soil texture, soil depth, fertilizer application, rainfall, plant growth, agricultural management, nutrient and water holding capacity (Sitienei et al., 2016; Yang et al., 2021; Liu & Han, 2021; Galindo, Strock & Pagliari, 2022). The characterization of the spatial distribution of soil nutrients is essential to a better understanding of the relationships between soil properties and environmental factors. In recent years, cultivated soil is related to the quality of crops, and its quality has been widely concerned (Song & Pijanowski, 2014; Yu et al., 2020). With the continuous development of agricultural planting technology, the nutrients of cultivated land will change differently (Reddy et al., 2016; Bai & Zhang, 2018; AbdelRahman, Hegab & Yossif, 2021). The spatial distribution of various soil nutrients also shows certain variability characteristics. Quantitative research on these variability characteristics can better reveal the spatial distribution of various soil nutrients. Meanwhile, combined with the surplus and deficiency of various soil nutrients, the soil productivity can be better explained (De Oliveira & Rotta, 1973; Liu et al., 2018). In addition, reasonable analysis of the spatial variability of soil nutrients and the objective evaluation of soil fertility may effectively reveal the spatial distribution of soil nutrients and the status of soil fertility in each region. This can also provide scientific guidance for regional soil nutrient management, cultivated land monitoring, fertilizer loss and prevention of cultivated land quality degradation. It is of great significance to promote the quantitative research of soil science and the implementation of precision agriculture (Lalitha et al., 2021). On this basis, taking Xuwen County as the research object, this paper comprehensively analyzed the soil characteristics of cultivated land in Xuwen County by using geostatistics, GIS technology and fuzzy mathematics, and referring to the cultivated land quality grade standard (GB/T 33469-2016). Meanwhile, the soil fertility of cultivated land in this region was comprehensively evaluated, and the spatial distribution of various soil nutrients and the spatial distribution of soil comprehensive fertility index were drawn by GIS software. This can provide theoretical basis for improving the utilization rate of chemical fertilizer, fine management of cultivated land and ecological environment in this region. This study may guide significance to the soil ecological construction, restoration of soil degradation, soil resources development and utilization in agriculture, agricultural soil management and other area.

## Materials and methods

### Overview of the study area

Xuwen County is located at the southernmost tip of China mainland, between 109°52′–110°35′E and 20°13′–20°43′N. The county is a low-hill platform terrain, and the terrain slopes from north to east, west and south along the coast. The north is higher, and the elevation is generally 100–150 m. It has a tropical monsoon climate with sufficient sunshine and abundant solar radiation. The annual average sunshine is 2078.7 h, and the annual average temperature is 23.3 °C. The four seasons are always green like spring. It is dry in winter and spring, while hot, rainy, thunderous and typhoon in summer and autumn. The surface water in the county is relatively poor, and the annual rainfall is uneven, accounting for 70% of the whole year from July to September. The average annual rainfall in the northeast is 1795 mm, and that in the southwest coast is 1364.1 mm. The average annual runoff depth is 466 mm, and the surface water runoff is 690 million cubic meters. The soil can be divided into 12 orders, 63 suborders, 250 great groups, 1400 sub-groups, 8000 families and 19000 series (Soil Taxonomy), among which laterite soils and salty soils soil are the main ones in the county (Soil Survey Staff & Soil Taxonomy, 1999). The geological structure of Xuwen County is relatively single, and the Quaternary volcanic eruption product basalt covers almost the whole county. Soil material at study location contains more amounts of organic carbon above for mineral soil material and hence it is considered organic soil material according to USDA soil taxonomy (Bockheim et al., 2014).

### Sample collection and analysis of evaluation index and determination of weight

**Figure 1:**
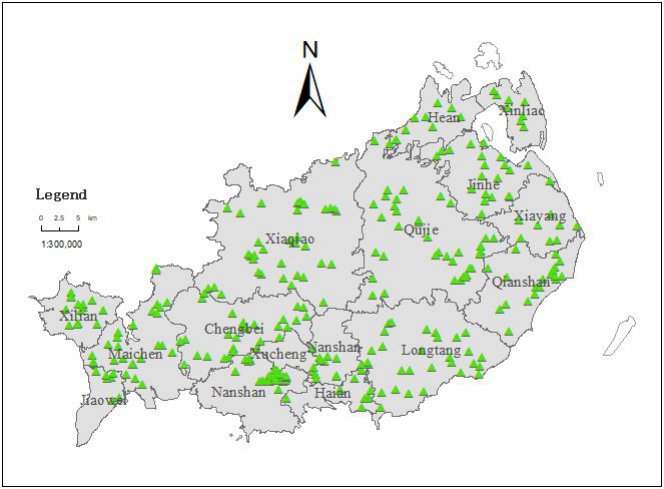
Sampling sites in the study area.

Following the principle of representativeness and uniformity, and combined with the status quo of land use, landform units and vegetation, the soil sampling points were arranged according to the cultivated land in Xuwen County. Total 300 sampling points were arranged (Fig. 1), and handheld GPS positioning was used to record the latitude, longitude, and elevation of the sampling points. Taking the locating point as the center, 4 surface soil samples (0–20 cm) were randomly selected in a circular area with a radius of 10 m for mixing, and then about 1 kg soil samples were taken by quartering method to the laboratory for air drying, grinding, and screening.

In addition, glass electrode method was used to determine soil pH, potassium dichromate oxidation method to determine soil organic matter (SOM) content, alkaline hydrolysis diffusion method to determine available nitrogen (AN) content, molybdenum antimony anti-colorimetric method to determine available phosphorus (AP) content, and flame photometric method to determine the content of available potassium (AK) (Lu, 2002).

### Construction of membership function for evaluation tabulation

In this paper, fuzzy mathematics principle is used to establish the corresponding membership function for each soil fertility index, and the membership value is calculated to express the state value of each fertility index. Fuzzy mathematics is used previously in many studies as reported earlier (Dong et al., 2011; Zhao et al., 2014). The abundance and deficiency indicators of single fertility vary greatly due to different soils and crops. According to the previous results (Nie et al., 2016) and Luo Bosheng’s research on numerical comprehensive evaluation of soil fertility, the effect curve of soil pH, SOM, AN, AP, and AK on crops presents S-type in a certain range. Based on the previous results and the actual production of local crops, this paper uses the S-curve membership function to study the soil in Xuwen County. The function is shown in formula (1). For the convenience of calculation, the curve function is transformed into the corresponding broken line function.

(1) \begin{eqnarray*}f(x)= \left\{ \begin{array}{@{}l@{}} \displaystyle 1.0 x\geq {x}_{2}\\ \displaystyle 0.9(x-{x}_{1})/({x}_{2}-{x}_{1})+0.1 {x}_{1}\leq x\lt {x}_{2}\\ \displaystyle 0.1 x\lt {x}_{1}\\ \displaystyle \end{array}. \right. \end{eqnarray*}

Since each individual fertility deficit index is different for different crops and soils, according to the research object of this article, and combined with the specific actual conditions of the brick red soil, the corresponding values are determined (Yageta et al., 2019) as shown in Table 1.

**Table 1:** Turning point values of soil fertility indexes.

Index	Turning point	
	*x*_1_	*x*_2_
pH	4.5	5.5
SOM/g kg^−1^	5	20
AN/mg kg^−1^	30	120
AP/mg kg^−1^	20	90
AK/m g kg^−1^	60	150

**Notes.**

SOM, soil organic matter; AN, alkali-hydrolyzable N; AP, available P; AK, available K.

**Figure 2:**
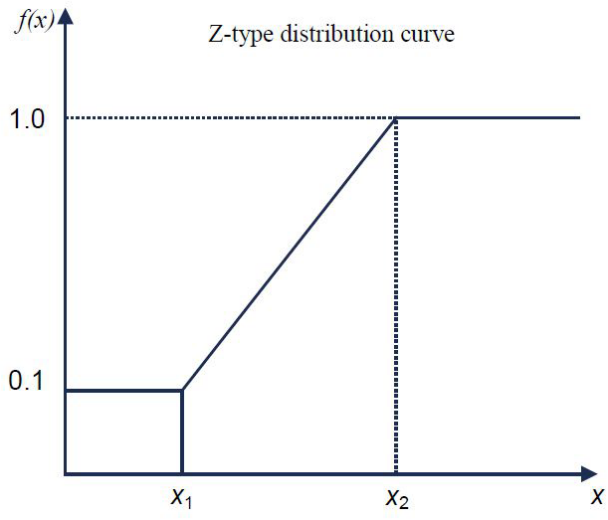
Membership function model and empirical formula. X is the measured value of the evaluation index, X1 is the lower limit of the evaluation index and X2 is the upper limit of the evaluation index.

Note: X in the Fig. 2 is the measured value of the evaluation index, X1 is the lower limit of the evaluation index and X2 is the upper limit of the evaluation index.

### Calculation of comprehensive evaluation index

This paper uses the weighted summation of weights in fuzzy mathematics to calculate the Integrated Fertility Index (IFI), the expression is as follows:

(2) \begin{eqnarray*}\mathrm{IFI}={\mathop{\sum \nolimits }\nolimits }_{\mathrm{i}=1}^{\mathrm{n}}({\mathrm{f}}_{\mathrm{ i}}\times {\mathrm{w}}_{\mathrm{i}})\end{eqnarray*}

where, n is the participating indicators; f_i_ is the membership degree of the i-th index, and W_i_ is the weight of the i-th index. Since the range of membership degree is [0.1, 1.0], and the IFI value is also [0.1, 1.0], thus the closer the value is to 1, the higher the soil fertility.

### Data processing

The analysis used the threshold method to identify the peculiar value. The peculiar value is the value which was in the range of the mean value that means plus or minus three times the standard deviation is regarded as the normal value, and the value outside the range is regarded as the peculiar value (Nouy, Chevreuil & Safatly, 2011; Roul & Madduri, 2018). The logarithm or the square root was converted to a normal distribution or an approximate normal distribution for all those data that does not obey the normal distribution, and finally the converted data was subjected to spatial analysis (Yang et al., 2020).

### Statistical analysis

This paper uses Excel 2010 and SPSS 20.0 software to conduct descriptive analysis and normality test on the sample data. The geostatistics module of ArcGIS 10.7 software is used to conduct Semivariance Analysis on the data. Its Kringing interpolation method interpolates each element to make a spatial distribution of soil nutrients and soil comprehensive fertility index. In addition, the data of pH and ANare logarithmically converted, and the data of SOM, AP and AN are square root converted. After K-S test, it is found that the data accord with normal distribution (Table 2) and meet the requirements of semi variance function analysis.

**Table 2:** Statistical parameters of the factors.

Index	Data transformation	Mean	SD	Kurtosis	Skewness	Frequency distribution
pH	LOG	0.74	0.22	−0.8009	0.1735	Normal distribution
SOM /g kg^−1^	SQRT	5.32	1.42	−0.1249	−0.1123	Normal distribution
AN /mg kg^−1^	LOG	2.11	0.18	0.4997	−0.3926	Normal distribution
AP /mg kg^−1^	SQRT	9.03	3.34	−0.4300	0.3361	Normal distribution
AK /mg kg^−1^	SQRT	13.90	4.89	−0.7209	0.0334	Normal distribution

**Notes.**

SOM, soil organic matter; AN, alkali-hydrolyzable N; AP, available P; AK, available K.

## Results

### Descriptive statistical analysis of soil nutrients in cultivated land

It can be seen from Table 3 that the average content of soil pH, SOM, AN, AP and AK in Xuwen County are 5.56, 30.33 g kg^−1^, 140.70 mg kg^−1^, 92.63 mg kg^−1^ and 217.08 mg kg^−1^, respectively. In addition, according to the nutrient classification standard of the second national soil survey (Tang, 1989), it is found that the average content of AP is higher, and the content of other nutrients is appropriate. The coefficient of variation (CV) can explain the spatial variability of soil characteristics. The grades are CV<10%, 10% ≤CV ≤100%, and CV>100%, indicating weak, medium, and strong variability respectively (Yang et al., 2013). It can be seen from Table 4 that the CV of soil nutrients in cultivated land of Xuwen County is: AP>AK>SOM>AN>pH, all of which belong to medium variation. Among them, the highest coefficient of variation of AP is 70.42%, while the lowest coefficient of variation of soil pH is 18.57%.

**Table 3:** Statistical characteristic values of soil nutrients.

Indexes	Sample sites	Min.	Max.	Mean	**SD**	C.V./%
pH	300	3.69	8.35	5.56	1.03	18.57
SOM/g kg^−1^	300	0.53	74.11	30.33	15.10	49.77
AN/mg kg^−1^	300	34.80	306.43	140.70	55.15	39.19
AP/mg kg^−1^	300	5.47	288.69	92.63	65.23	70.42
AK /mg kg^−1^	300	10.96	634.85	217.08	139.02	64.04

**Notes.**

SOM, soil organic matter; AN, alkali-hydrolyzable N; AP, available P; AK, available K; C.V., coefficient of valiation.

**Table 4:** Semi-variance function theory model and parameters of soil nutrients.

Index	Model	Main variation/km	Variation /km	ARC	Azimuth	C_0_	C_0_+C	C_0_/(C_0_+C)	MSE
pH	Gaussian	0.1390	0.0886	1.5689	136.5820	0.0225	0.0301	74.75%	−0.0211
SOM/g kg^−1^	J-Bessel	0.0100	0.0168	1.6884	85.4297	5.7297	10.5931	54.09%	−0.0054
AN/mg kg^−1^	J-Bessel	0.0138	0.0155	1.1198	169.2773	0.1031	0.1625	63.46%	−0.0308
AP /mg kg^−1^	Gaussian	0.0056	0.0034	1.6486	159.2578	28.9266	46.4726	62.24%	0.0031
AK /mg kg^−1^	J-Bessel	0.0615	0.0397	1.5476	78.9258	61.9723	76.1515	81.38%	−0.0168

**Notes.**

ARC, anisotropy ratio coefficient; C_0_, the nugget; C_0_+C, abutment value; MSE, normalized mean error.

### Spatial structure analysis of soil nutrients in cultivated land

The geostatistics module of ArcGIS 10.7 software is used for semi variance analysis of the data. Comparing the results of each interpolation model, the interpolation model with the standard average value closest to 0, the smallest root means square prediction error, the average standard error closest to the root mean square prediction error, and the standard root mean square prediction error closest to 1 is selected. The results (Table 4) find that the optimal interpolation model for soil pH and AP in Xuwen County is the Gaussian model, and the optimal interpolation model for the remaining three indicators is the J-Bessel model. Further analysis shows that the content of soil pH, SOM, AN, AP and AK all have spatial autocorrelation in a certain range, but the specific performance is different. From the nugget value of C_0_, the order of the nugget value is AK>AP>SOM>AN>pH. The maximum C_0_ of AK is 61.9723, indicating the largest variation caused by random factors. The minimum C_0_ of pH is 0.0225, indicating that the spatial variation caused by random factors, such as management, sampling and testing is small. From the point of view of C0/(C0+C) value of the block base ratio, the order of the block base ratio is AK>pH>AN>AP>SOM. The ratio of soil AK is greater than 75%, the spatial autocorrelation is weak, and the spatial variability is greatly affected by random factors. The block basis ratio of soil pH value, SOM, AN and AP is between 25–75%.

### Spatial distribution characteristics of soil nutrients in cultivated land

**Figure 3:**
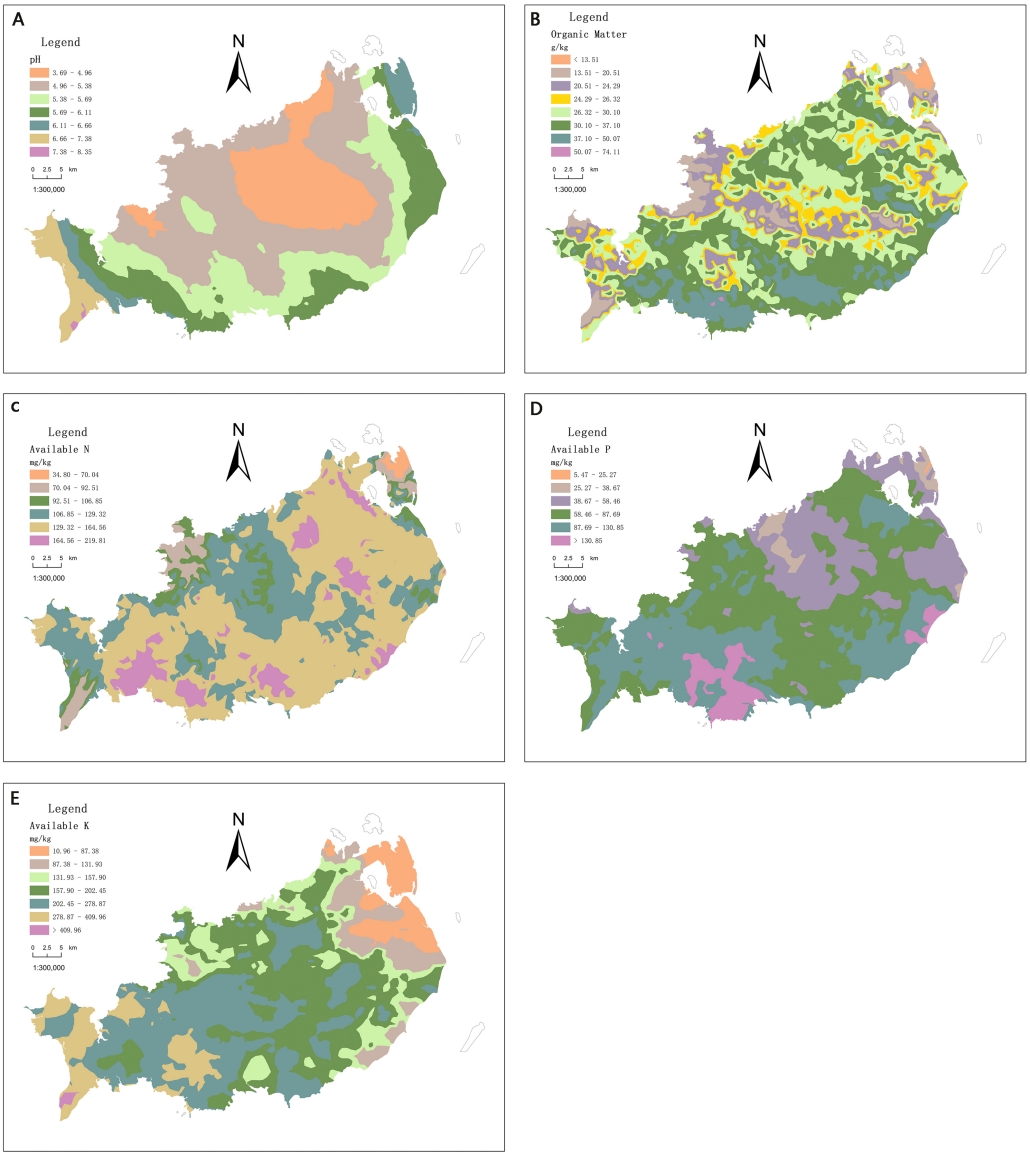
Distribution of (A) pH, (B) SOM, (C) AN, (D) AP and (E) AK.

To display the spatial distribution characteristics of various soil nutrients more intuitively, Kriging interpolation is carried out for each soil nutrient by using Arcgis software. The obtained spatial distribution of soil pH, SOM, AN, AP and AK content is shown in Fig. 3. The soil pH value of cultivated land in Xuwen County is in 3.69–8.35. There are very few alkaline spots (pH value greater than 7.5) in this area, only accounting for 4.33% and mainly acidic spots (Fig. 3A). The organic matter is rich in 0.53–74.11 g kg^−1^, with an average of 30.33 g kg^−1^. Among them, the average content of SOM in plough layer of Hai’an town and Xucheng town is higher than the average of the county with 30.33 g kg^−1^. The organic matter of cultivated land surface soil in Jiaowei town and Xinliao town is lower than the average (Fig. 3B). The content of AN in the area is in 34.8–497.9 mg kg^−1^, with an average of 34.8–497.9 mg kg^−1^. Its content is obviously different for different regions. Among them, the highest is 201.48 mg kg^−1^ in Hai’an Town, followed by 179.94 mg kg^−1^ in He’an Town, and the lowest is 66.74 mg kg^−1^ in Xinliao Town (Fig. 3C). The AP is in 5.47–379.58 mg kg^−1^, with an average of 93.27 mg kg^−1^, and the overall content is relatively high. Among them, the average content of AP in the cultivated land of Xucheng Town is the highest, with 141.42 mg kg^−1^; Next is Nanshan Town, with 134.39 mg kg^−1^, and the lowest is Xinliao Town, with 30.57 mg kg^−1^ (Fig. 3D). The content of AK is in 10.96–759.65 mg kg^−1^, with an average of 217.62 mg kg^−1^. Among them, the average content of AK of cultivated land in Jiaowei Town and Xucheng Town ranks in the forefront, with 340.50 mg kg^−1^ and 330.67 mg kg^−1^, respectively. The average content of AK in the cultivated land of Xinliao Town and Jinhe Town is 48.73 mg kg^−1^ and 96.42 mg kg^−1^, respectively (Fig. 3E).

### The spatial distribution of soil comprehensive fertility index (IFI)

**Figure 4:**
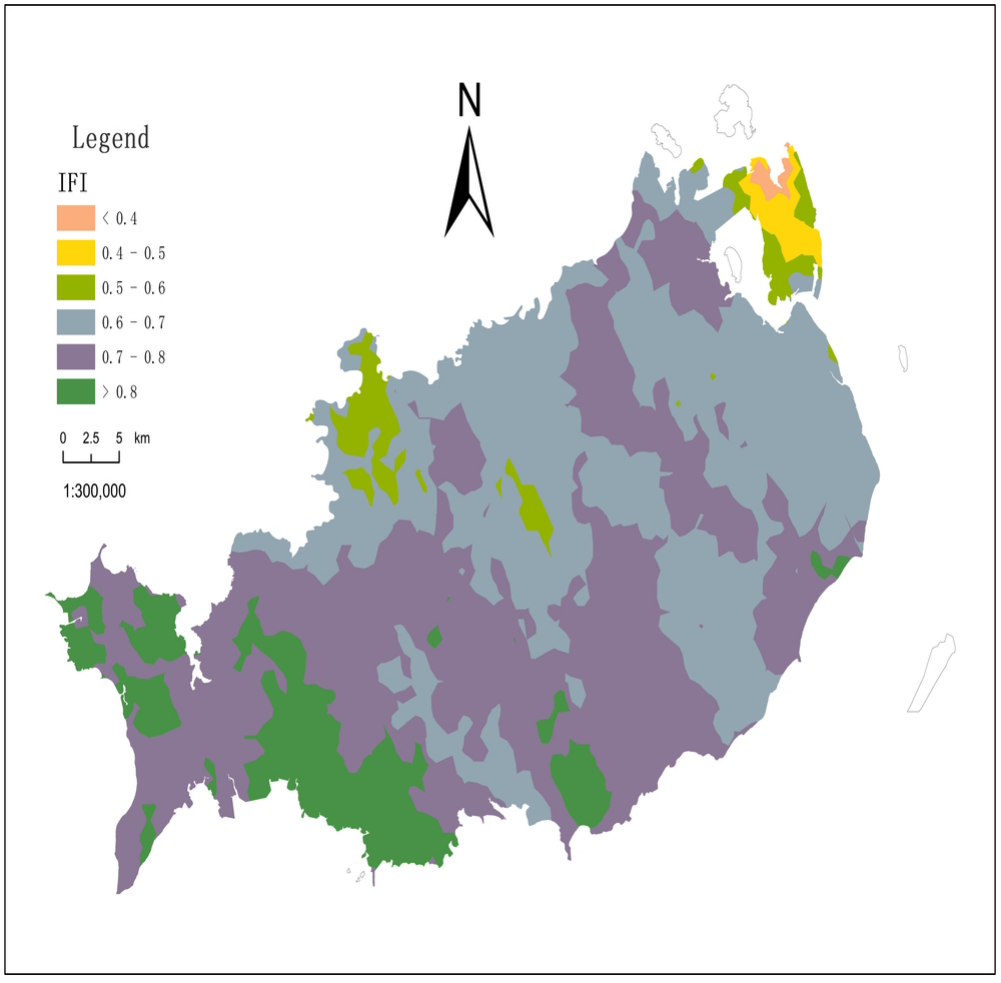
Spatial distribution of comprehensive soil fertility index in Xuwen County.

The spatial distribution of soil comprehensive fertility index in Xuwen County is drawn by Kriging interpolation method (Fig. 4). As shown in Table 5, a statistical analysis of the IFI values of 300 sampling points show that the IFI values of samples are in 0.115–0.893, with an average of 0.457, and 32.31% of CV. Thus, the variation is moderate. After Arcs in transformation, the IFI value obeys the normal distribution. Comparing the various interpolation models, the optimal model of the IFI in the area is the spherical model. The ratio of the nugget value to the abutment value is 37.13%, showing a moderate spatial correlation. This implies that its spatial variability is affected by the combination of structural and random factors (Hosseini, Naeini & Bameri, 2016). The soil comprehensive fertility index (IFI) of Xuwen County is divided into five grades, which are excellent (>0.8), good (0.7–0.8), general (0.6–0.7), medium (0.4–0.6) and poor (<0.4).

## Discussion

The study area is located at the southernmost part of Chinese mainland. It is affected by high temperature and rainy monsoon climate all the year round, and the rainfall is more concentrated. The salt base leaching in the soil is strong, and the soil potassium element is easy to dissolve in water. It is easy to cause leaching loss (Ahmed et al., 2006; Islam et al., 2014), and make the reserves of soil potassium element in the area congenitally insufficient. However, the migration of AP in soil includes complex processes, such as diffusion, adsorption, desorption, deposition, and burial accumulation. The climate, parent material, and soil conditions in Xuwen County are basically the same, but the abundance and deficiency of various soil nutrient indicators were varied which is quite common everywhere. After long-term agricultural planting, the soil nutrients also have changed as reported earlier as well (Bommayasamy, Singh & Rahman, 2020). This study is to explore the spatial distribution characteristics of soil nutrients within the county, to understand the spatial changes of soil nutrients, evaluate the soil fertility of cultivated land, and provide a scientific basis for formulating reasonable land use management plans and technical support for precision agriculture.

### The variability of soil nutrients

Soil nutrient dynamics vary with time following afforestation, typically declining over the short-term (<20 years), but increase over the long-term, *via* atmospheric N deposition and biological nitrogen fixation. The extent that these drives might invoke subsequent consequences for soil sequestration remain uncertain and some of the mechanisms is still unknown. There were differences in various nutrient indicators. In addition to natural factors, such as climate, parent material and soil, they may also have a lot to do with agricultural activities, such as farming, planting methods and fertilization habits (Hosseini, Naeini & Bameri, 2016; Li et al., 2017; Singh et al., 2019). There was spatial variation in soil nutrient content in Xuwen County, and pH, SOM, AN, AP and AK all belonged to moderate variation. According to the nutrient classification standard of the “Second National Soil Census”, most of the nutrients in the soil samples in the study area were generally suitable, and the average content of available phosphorus was particularly high, but all belonged to moderate variation. The study area belongs to acidic red soil in southern China, which is rich in iron and aluminum ions. It is easy to chemically react with phosphate fertilizers applied to the soil and form sediments and accumulate in the soil, which cannot be used by plants, resulting in generally high soil available phosphorus content (Liao et al., 2019). However, different types of organic fertilizers and soil parent materials have different capacity to fix soil phosphorus, resulting in differences in soil available phosphorus content (Mehmood et al., 2015; Shu, Qi & Wang, 2021). Soil nutrient sources, utilization efficiency and their migration in soil are the main factors that determine the spatial variation of soil nutrients (Sun et al., 2018). According to the survey, rice, pineapples, and bananas are mainly planted in Xuwen area, with a wide planting area in Qujie, Qianshan, Maichen, Jinshan and Longtang. The main reason is that local growers pay more attention to the application of nitrogen and phosphorus fertilizer in the planting process, while ignoring the important impact of Potassium Fertilizer on crop growth. The difference between the minimum and maximum values of soil nutrient content in Table 4 is obvious, which indicates that there are background differences in soil nutrient content at each sample point. The blind average fertilization will cause continuous shortage of soil nutrients in low nutrient areas and the continuous surplus of soil nutrients in high nutrient areas. The formation mechanism of soil fertility is very complex, different scholars have different understandings. The evaluation methods and indicators are not consistent, and the evaluation of soil fertility is mainly based on the content of soil nutrients (Sindhu, Ravikumar & Shivakumar, 2019). According to the influence of various nutrient indicators on soil productivity and previous research (Mondal et al., 2016), this paper selects five indicators: soil pH, SOM, AN, AP and AK, to establish a soil fertility evaluation index system for Xuwen County. The weight of each index (Table 6) is calculated and determined by the analytic hierarchy process (El Hamzaoui, El Baghdadi & Hilali, 2021), and then the random consistency test index is verified that the matrix does not need to be adjusted.

### The spatial structure of soil nutrients

The spatial autocorrelation is medium, and the spatial variation is affected by structure and random factors. The variation range, also known as the spatial maximum correlation distance, reflects the sample spatial autocorrelation range (Li, Zhou & Zhang, 2013). The ratio of the main variable range to the secondary variable range is the anisotropy ratio coefficient. The order of variable range in the area is: pH>AK>SOM>AN>AP. The direction of the main range is northwest-southeast, and the direction of the main range of SOM and AK is east–west. it may be because the country is affected by high temperature and rainy monsoon climate all the year round, and the rainfall is more concentrated. Therefore, the content of organic matter and available potassium is greatly reduced, while organic matter and total nitrogen are the main sources of arable soil to provide nutrients for crops; at the same time, they directly affect the structure of the soil, the ability of soil to maintain and supply fertilizer, and the availability of other nutrients, *etc*. Studies have shown that the salt base leaching in the soil is strong, and the soil potassium element is easy to cause leaching loss (Ahmed et al., 2006; Islam et al., 2014). Small-scale heterogeneity of soil nutrient distribution It may be more affected by structural and random factors (such as chemical fertilizers, pesticides, and plant growth hormones, *etc*.). In addition, the fertilizer use efficiency of different plants is different, resulting in large spatial differences in the nutrients remaining in the soil (Nanganoa et al., 2020).

**Table 5:** The theoretical model of IFI semi-variance function and its parameters.

Index	Model	Variation		ARC	Azimuth	C_0_	C_0_+C	C_0_/(C_0_+C)	MSE
		Main variation	Variation						
IFI	Spherical model	0.0158	0.0104	1.5166	149.4141	0.0166	0.0448	37.13%	0.0196

**Notes.**

ARC, anisotropy ratio coefficient; C_0_, the nugget; C_0_+C, abutment value; MSE, normalized mean error.

**Table 6:** Weights of soil fertility indexes.

Indexes	pH	SOM/g kg^−1^	AN /mg kg^−1^	AP /mg kg^−1^	**AK** /mg kg^−1^
Weight	0.1460	0.2919	0.2257	0.1682	0.1682

**Notes.**

SOM, soil organic matter; AN, alkali-hydrolyzable N; AP, available P; AK, available K.

### The spatial distribution of soil nutrients

This paper uses ordinary kriging to interpolate it to analyze the spatial distribution characteristics of nutrients in soil, which can effectively reveal the relationship between soil elements and environmental variables, which has been proved in this study and other studies (Shao et al., 2012; Sun, Li & Du, 2017). From the distribution of each town, the cultivated soil samples in He’an Town are all acidic, and the proportion of acid samples in Qujie Town, Jinhe Town and Xiayang Town are all above 90%. Therefore, there are certain regional differences in the overall content. The soil pH is generally acidic; SOM and AN is rich; AP is generally high, and AK is generally low which is a clear difference in various nutrients occurrence. From the perspective of the phosphorus content of the cultivated land in each town, there are certain differences between different regions. Among them, the average content of AP in the cultivated land of Xucheng Town is the highest, with 141.42 mg kg-1; Next is Nanshan Town, with 134.39 mg kg-1, and the lowest is Xinliao Town, with 30.57 mg kg-1. The content of AK is in 10.96–759.65 mg kg-1, with an average of 217.62 mg kg-1. In view of the potassium of the cultivated land in each town, the overall content is low with large differences in regions. The spatial variation of soil nutrients is the result of the combined action of natural (climate, soil parent material, topography, soil physical and chemical properties, c) and human factors (fertilization, farming methods, planting structure, *etc*.) (Hosseini, Naeini & Bameri, 2016; Li et al., 2017; Singh et al., 2019; Bommayasamy, Singh & Rahman, 2020). The results of this study show that there are certain regional differences in the overall content. The soil pH is generally acidic; SOM and AN is rich; AP is generally high, and AK is generally low which is a clear difference in various nutrients occurrence. The study area Located in the coastal zone of southern China, the main soil is basalt parent material, and the soil types are mainly clay and loam. Rice is grown in many paddy fields, and dry crops such as pineapples and bananas are grown on dry land. Studies have shown that parent material has the most significant impact on soil formation and is generally significantly correlated with soil nutrients (Qiu et al., 2016; Ke et al., 2018; Xu et al., 2019). Zhong et al. (2019) found that soil-forming parent materials can affect the accumulation and decomposition of nitrogen in the soil, and clays developed from parent materials such as sediments, limestone, mudstone, and basalt have high organic nitrogen storage capacity. Similarly, Li et al. (2021) found that the natural factors that determine soil potassium variability are climate >terrain >mineral composition >soil physicochemical properties. The impact of human activities on soil nutrients is mainly manifested in agricultural production, spatial distribution, and ecological environment. Different fertilization methods and land use methods will affect the spatial distribution of soil nutrients. Sun, Li & Du (2017) used GIS technology to study the spatial variability and influencing factors of soil nutrients. This study only carried out spatial variation analysis and soil fertility evaluation of soil nutrients in the study area. In the future, the influence of different factors on the spatial distribution of soil nutrients can be explored.

### The spatial distribution of soil fertility

The formation mechanism of soil fertility is very complex, and the evaluation methods and indicators are not consistent, but the evaluation of soil fertility is mainly based on the content of soil nutrients (Sindhu, Ravikumar & Shivakumar, 2019; Liu et al., 2006). According to the influence of various nutrient indicators on soil productivity and previous research (Mondal et al., 2016), this paper selects five indicators: soil pH, SOM, AN, AP and AK, to establish a soil fertility evaluation index system for Xuwen County. The weight of each index (Table 6) is calculated and determined by the analytic hierarchy process (El Hamzaoui, El Baghdadi & Hilali, 2021), and then the random consistency test index is verified that the matrix does not need to be adjusted. The results of spatial distribution analysis of soil comprehensive fertility index in Xuwen County show that the quality of cultivated land is at the upper middle level. The indexes with excellent comprehensive fertility index account for 26.00%, the good indexes account for 32.67%, the general indexes account for 19.33%, the medium indexes account for 19.00%, and the poor indexes account for 3.00%. Specifically, for areas with high soil fertility in the study area, excessive fertilization should be avoided, and chemical fertilizer additions can be reduced by reducing fertilization, soil testing and formula fertilization, and applying organic fertilizers instead of chemical fertilizers (Li-Zhong et al., 2015; Sun, Li & Du, 2017; Dolobeshkin, Gumbarov & Bandurin, 2021). For some areas with low soil fertility, the agricultural planting structure and farming methods should be adjusted according to the nutrient status, and measures such as reasonable fertilization and application of soil amendments should be formulated to improve soil fertility and change the uneven spatial distribution of regional nutrients (Krauss et al., 2020).

The accumulation and decomposition of organic matter can directly affect the transformation and storage of nitrogen. In addition, the mineralization of organic matter can also release AN. The spatial autocorrelation is medium, and the spatial variation is affected by structure and random factors. The variation range, also known as the spatial maximum correlation distance, reflects the sample spatial autocorrelation range (Li, Zhou & Zhang, 2013). The ratio of the main variable range to the secondary variable range is the anisotropy ratio coefficient. The order of variable range in the area is: pH>AK>SOM>AN>AP. The direction of the main range is northwest-southeast, and the direction of the main range of SOM and AK is east–west. The results of spatial distribution analysis of soil comprehensive fertility index in Xuwen County show that the quality of cultivated land is at the upper middle level. The indexes with excellent comprehensive fertility index account for 26.00%, mainly distribute in Maichen Town, Qujie Town, Nanshan Town, and Chengbei town. The good indexes account for 32.67%, mainly distribute in Chengbei Town, Longtang Town, Maichen Town, and Jinhe town. The general indexes account for 19.33%, mainly distribute in Xiaqiao Town, Qujie Town, Chengbei Town, and Nanshan Town. The medium indexes account for 19.00%, mainly distribute in Xiaqiao Town, Qujie Town, Longtang Town, and Qianshan Town. The poor indexes account for 3.00%, mainly distribute in Xinliao Town and Xiaqiao Town.

## Conclusion

The variation coefficients of five soil nutrients in the area are in 18.57–70.42%, and the order is: AP >AK >SOM >AN>pH, and all are medium variation. The optimal interpolation model of soil pH and AP in the area is Gaussian model, while the optimal interpolation model of SOM, AN and AK is J-Bessel model. The soil in the study area is basically acidic, and the content of AP is relatively high. The content of other nutrients is appropriate, and the spatial correlation of AK is weak, while other indicators show moderate spatial correlation. The soil fertility quality in the area shows an obvious trend of high in the South and low in the North. The overall level of soil fertility is medium to upper. The soil with better fertility is mainly distributed in Maichen Town, Qujie Town, Nanshan Town, Chengbei Town, Longtang Town, and Jinhe town. The proportion of excellent, good, general, medium, and poor comprehensive fertility indexes are 26.00%, 32.67% and 19.33%, 19.00% and 3.00%, respectively. Therefore, from the spatial distribution of nutrients, the distribution patterns of SOM and AN in the study area are similar. Thus, in the future agricultural production, we should achieve balanced fertilization, timely supplement the application of potassium fertilizer, apply organic fertilizer and nitrogen fertilizer in a planned way, and strictly control phosphorus fertilizer to avoid soil hardening caused by excessive phosphorus fertilizer. In summary, there are mechanisms that may increase or decrease soil nutrient availability in soils, and this is an area that requires much more investigation.

##  Supplemental Information

10.7717/peerj.13239/supp-1 Supplemental Information 1 Raw data applied for data analyses and preparation for Figs. 1 and 3 and Tables 1–4

10.7717/peerj.13239/supp-2 Supplemental Information 2 The proportion of various nutrients (including pH value, organic matter, alkali hydrolyzable nitrogen, available phosphorus and available potassium) of 300 soil samples in the soil quality

10.7717/peerj.13239/supp-3 Supplemental Information 3 The contents were determined for pH value, organic matter, alkali hydrolyzable nitrogen, available phosphorus and available potassium of 300 soil samples in the survey area

## Additional Information and Declarations

### Data Availability

The following information was supplied regarding data availability: The raw data are available in the Supplemental Files.
